# World TB Day — March 24, 2014

**Published:** 2014-03-21

Each year, World TB Day is observed on March 24. This annual event commemorates the date in 1882 when Dr. Robert Koch announced his discovery of *Mycobacterium tuberculosis*, the bacillus that causes tuberculosis (TB). World TB Day provides an opportunity to raise awareness about TB-related problems and solutions and to support worldwide TB control efforts. For 2014, CDC selected the theme “Find TB. Treat TB. Working together to eliminate TB.” Health officials in local and state TB programs are encouraged to reach out to their communities to raise awareness about TB and partner with others who are caring for those most at risk for TB. Everyone has a role in ensuring that one day TB will be eliminated.

In 2013, a total of 9,588 new TB cases were reported in the United States, for a rate of 3.0 cases per 100,000 (1). Although the number of TB cases continues to decline, challenges remain that slow progress toward the goal of TB elimination in the United States. TB still persists at greater incidence rates in specific populations. Foreign-born persons and racial/ethnic minorities continue to be affected disproportionately.

CDC is committed to a world free of TB. Initiatives to improve awareness, testing, and treatment of latent TB infection and TB disease among high-risk groups are critical to reaching the goal of TB elimination in the United States. Additional information about World TB Day and CDC’s TB elimination activities is available at http://www.cdc.gov/tb/events/worldtbday.
